# Amelioration of Lupus Serum-Induced Skin Inflammation in CD64-Deficient Mice

**DOI:** 10.3389/fimmu.2022.824008

**Published:** 2022-02-22

**Authors:** Lijuan Jiang, Xiaoxiao Han, Wenlin Qiu, Tong Yu, Ruizhi Feng, Xuefei Wang, Xiaoru Duan, Guo-Min Deng

**Affiliations:** Department of Rheumatology and Immunology, Wuhan Union Hospital, Tongji Medical College, Huazhong University of Science and Technology, Wuhan, China

**Keywords:** systemic lupus erythematosus, monocytes/macrophages, CD64, flow cytometry, inflammation

## Abstract

Systemic lupus erythematosus (SLE) is a heterogeneous autoimmune disorder characterized by high autoantibodies levels and multiorgan tissue damage. The current study investigated the role of CD64 in SLE patients and animal models. According to a flow cytometry study, SLE patients showed an increase in CD64 expression in circulating monocytes. There was a correlation between CD64 and SLEDAI, blood urea nitrogen levels, and anti-Sm antibodies. In skin lesions of lupus MRL/*lpr* mice, there was high IgG deposition and CD64 expression. *In vitro*, cytokines IL-10 and IFN-γ upregulated CD64 expression in monocytes/macrophages that was inhibited by glucocorticoids. In CD64-deficient mice, skin inflammation induced by lupus serum was reduced. Furthermore, activation of spleen tyrosine kinase (Syk), Akt, and extracellular signal-regulated kinase (Erk) was inhibited in CD64-deficient monocytes. The results suggest that CD64 could be a biomarker for observing SLE progression, as well as a mechanistic checkpoint in lupus pathogenesis.

## Introduction

Systemic lupus erythematosus (SLE), an autoimmune disease with a complex pathophysiology and clinical manifestations, lacks specific prognostic indicators (1, 2). SLE is characterized by high autoantibodies levels (anti-dsDNA, anti-Sm, and anti-phospholipid antibodies) in serum and certain clinical manifestations such as skin inflammation and lupus nephritis (3, 4). Treatment of SLE with glucocorticoids, hydroxychloroquine, and immunomodulators (methotrexate, azathioprine, mycophenolate) has been recommended as the first-line treatment (5, 6).

FcγRs are receptors for the constant (Fc) region of IgG and are extensively expressed on the membrane of immune cells. CD64 (FcγRI) is the only known high-affinity FcγR for IgG with a restricted isotype specificity, whereas IgG affinity of CD32 (FcγRII) and CD16 (FcγRIII) is comparatively lower (7, 8). Activating FcγRs with immunoreceptor tyrosine-based activation motif (ITAM) in intracellular structure, CD64 and CD16 recruit spleen tyrosine kinase (Syk), which assembles into complexes at cell membrane *via* interaction between its SH2 domains and the receptor tyrosine-phosphorylated ITAM domains (9).

Phosphoinositide 3-kinase (PI3K)/Akt (Protein Kinase B) signaling pathway is firmly linked with Syk (10–12). Akt, a mechanistic target of rapamycin complex 2 (mTORC2) substrate, regulates various cellular responses through phosphorylation and inactivation (13). Mitogen-activated protein kinases (MAPKs) are serine/threonine-specific kinases family, comprising extracellular signal-regulated kinase (Erk) and c-Jun N-terminal kinase (JNK), which regulates cell proliferation, difference, motility, and death (14). Although PI3K/Akt and MAPKs signaling pathways are important in cellular responses, aberrant activation can result in inflammatory diseases (15).

Nuclear factor-kappa B (NF-κB), a promptly inducible transcription factor, is comprised of homo- and heterodimers of Rel A (p65), Rel B, p50, p52, and c-Rel in mammals (16). The NF-κB pathway modifies cell’s biology and is generally involved in multiple cell responses resulting from its hundreds of target genes. Activation of NF-κB is obtained through phosphorylation and degradation of IκB proteins (including IκBα), resulting in the release of NF-κB dimers (17).

Several of our previous studies indicate that infiltrated inflammatory cells, specifically monocytes, contribute to the pathogenesis of tissue injuries in SLE (18–22). Furthermore, we found that an animal model of lupus serum-induced skin inflammation is a useful tool to investigate the pathogenesis of skin injuries in SLE (19, 23). Herein, we studied the role of FcγRI/CD64 in the pathogenesis of lupus by analyzing the expression of CD64 in circulating monocytes from SLE patients and using a model of lupus serum-induced skin inflammation in CD64-deficient mice.

## Results

### CD64 Expression in Monocytes Increased in SLE Patients

**Figure 1:**
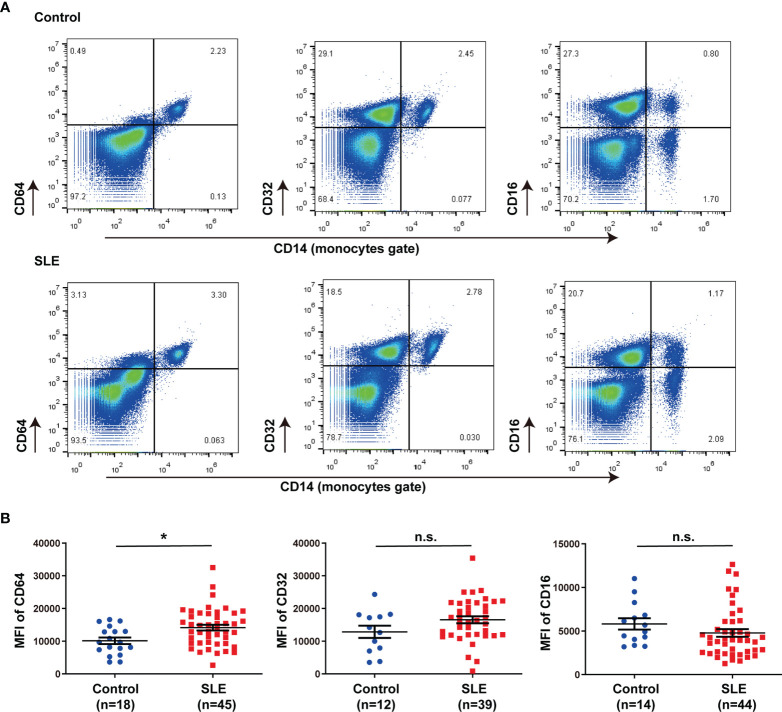
CD64 expression in monocytes increased in SLE. **(A)** Representative flow cytometry of CD64, CD32 and CD16 expression on CD14^+^ monocytes of peripheral blood mononuclear cells from SLE patients and healthy controls. **(B)** Flow cytometry analysis of surface expression of CD64, CD32 and CD16 on circulating monocytes in healthy controls and SLE patients. Detection of CD64 expression comprises 18 healthy controls (male:female=2:16, age 40.33±3.03) and 45 SLE patients (male:female=5:40, age 40.78±2.21). CD32 data comprises 12 healthy controls (male:female=2:10, age 43.25±4.12) and 39 SLE patients (male:female=3:36 , age 41.31±2.35). CD16 data comprises 14 healthy controls (male:female=2:12, age 42.79±3.61) and 44 SLE patients (male:female=3:41, age 40.31±2.14). Bars represent the average mean fluorescent intensity (MFI) of CD64, CD32 or CD16 on monocytes. Error bars represent standard deviation. *p < 0.05; ns, not significant.

In this study, we isolated peripheral blood mononuclear cells (PBMCs) from 45 SLE patients and 18 healthy controls and marked circulating monocytes with CD14. Data were collected on demographic, clinical characteristics, and medication (**Table 1**). The proportion of CD64^+^CD14^+^ and CD32^+^ CD14^+^ cells in PBMCs was about 3%, which accounts for almost all monocytes, but the proportion of CD16^+^ CD14^+^ cells was only about 1% in healthy controls and SLE patients (**Figure 1A**). CD64 expression was significantly increased in SLE patients compared with controls (**Figure 1B**), but CD32 and CD16 expression did not differ significantly (**Figure 1B**). These results revealed that CD64 was highly expressed on monocytes, and the CD64 expression increased in SLE patients.

**Table 1:** Demographics clinical characteristics and medication use of subjects.

	Controls (n = 18)	SLE (n = 45)
Male:Female	2:16	5:40
Age (years)	40.33 ± 3.03	40.78 ± 2.21
SLE manifestation (%)^a^		
CNS	–	22.22
Skin	–	42.22
Joint	–	20.00
Anti-dsDNA	–	46.51^b^
Anti-Sm	–	42.86^c^
Anti-phospholipid	–	11.63^b^
Medication use (%)		
Glucocorticoids	–	95.56
Antimalarials	–	55.56
Cyclophosphamide	–	26.67
Mofetil mycophenolate	–	25.49

a SLE manifestations were recorded at any point during the course of the disease.

b 43 of 45 SLE patients were tested anti-dsDNA and anti-phospholipid.

c 42 of 45 SLE patients were tested anti-Sm. SLE, systemic lupus erythematosus; CNS, central nervous system.

### CD64 Expression Was Correlated With SLE Activity

**Figure 2:**
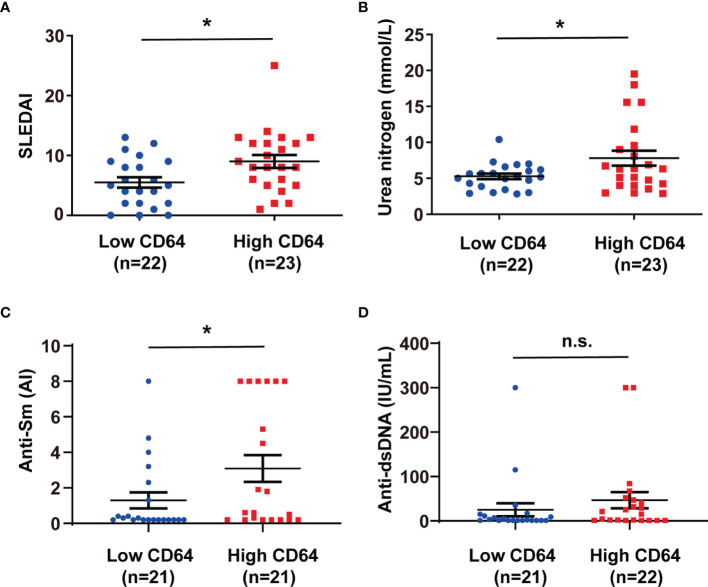
CD64 expression in monocytes correlated with SLEDAI, anti-Sm antibodies and blood urea nitrogen levels in SLE. SLE patients were classified into low and high CD64 groups, and the analysis of their SLEDAI **(A)**, blood urea nitrogen **(B)**, anti-Sm antibodies **(C)** and anti-dsDNA antibodies **(D)** were presented. 21 of 22 low CD64 patients were tested anti-dsDNA and anti-Sm; 21 of 23 high CD64 patients were tested anti-Sm; 22 of 23 high CD64 patients were tested anti-dsDNA. *p < 0.05; n.s., not significant.

To understand the potential role of dysregulation of CD64 in SLE, we estimated the relationship between CD64 expression on monocytes and SLE disease. In **Table 2**, clinical and laboratory data demonstrate the difference between high CD64 expression and low CD64 expression among patients with SLE. Patients with greater CD64 expression received a higher SLE disease activity index (SLEDAI) (**Figure 2A**). Furthermore, there were higher blood urea nitrogen levels, the indicator of nephritis, in high CD64 group patients (**Figure 2B**). Due to the important role of autoantibodies in the pathophysiology of SLE, we wondered if they have a correlation with CD64 expression. We certainly found a parallel upregulation of anti-Sm antibodies (**Figure 2C**). However, the elevation of anti-dsDNA antibodies was not significant (**Figure 2D**). These results showed that CD64 expression is related to lupus disease activity.

**Table 2:** Comparisons of CD64 expression (MFI) with laboratory measurements and dose of glucocorticoids in SLE.

	Low CD64 patients (n = 22)	High CD64 patients (n = 23)	*P* valve
MFI of CD64	9481 ± 588.7	18600 ± 897.3	0.0464*
SLEDAI	5.5 ± 0.86	9.0 ± 1.09	0.0161*
C3 (g/L)	0.58 ± 0.05^a^	0.60 ± 0.04^b^	0.7211
C4 (g/L)	0.14 ± 0.02^a^	0.12 ± 0.01^b^	0.3747
IgG (g/L)	14.61 ± 1.50	13.44 ± 1.09^c^	0.5333
Anti-dsDNA (IU/mL)	25.19 ± 14.80^a^	46.77 ± 18.21^c^	0.3656
Anti-Sm (AI)	1.30 ± 0.45^a^	3.09 ± 0.76^c^	0.0482*
CRP (mg/L)	6.08 ± 1.95^d^	7.13 ± 1.44^b^	0.6614
ESR (mm/h)	22.05 ± 4.89	26.09 ± 5.35	0.5810
Urea nitrogen (mmol/L)	5.27 ± 0.38	7.80 ± 1.04	0.0303*
Creatinine (mmol/L)	57.39 ± 3.43	65.34 ± 5.34	0.2215
IL-2 (pg/mL)	1.86 ± 0.18	1.50 ± 0.24^e^	0.2264
IL-4 (pg/mL)	1.67 ± 0.09	1.82 ± 0.08^e^	0.2439
IL-6 (pg/mL)	6.63 ± 0.87	25.64 ± 11.05^e^	0.0796
IL-10 (pg/mL)	3.65 ± 0.34	4.55 ± 1.35^e^	0.5040
TNF-α (pg/mL)	12.43 ± 4.34	11.41 ± 4.04^e^	0.8650
IFN-γ (pg/mL)	2.35 ± 0.49	6.43 ± 4.80^f^	0.3683
Dose of GCs (mg/d)	28.55 ± 6.11	38.12 ± 5.78	0.2612

According to the surface CD64 expression in circulating monocytes, SLE patients were classified into low and high CD64 groups. Laboratory measurements and dose of GCs (converted into prednisone) were showed by mean ± standard error of the mean (SEM).

a 21 of 22 low CD64 patients were tested C3, C4, anti-dsDNA and anti-Sm;

b 21 of 23 high CD64 patients were tested C3, C4, CRP and anti-Sm;

c 22 of 23 high CD64 patients were tested IgG and anti-dsDNA.

d 18 of 22 low CD64 patients were tested CRP;

e 20 of 23 high CD64 patients were tested IL-2, IL-4, IL-6, IL-10, TNF-α;

f 19 of 23 high CD64 patients were tested IFN-γ. *P < 0.05; P < 0.05 is considered statistically significant. Glucocorticoids, GCs.

### Large Amount of CD64 Expression in Skin Lesions in MRL/*lpr* Mice

**Figure 3:**
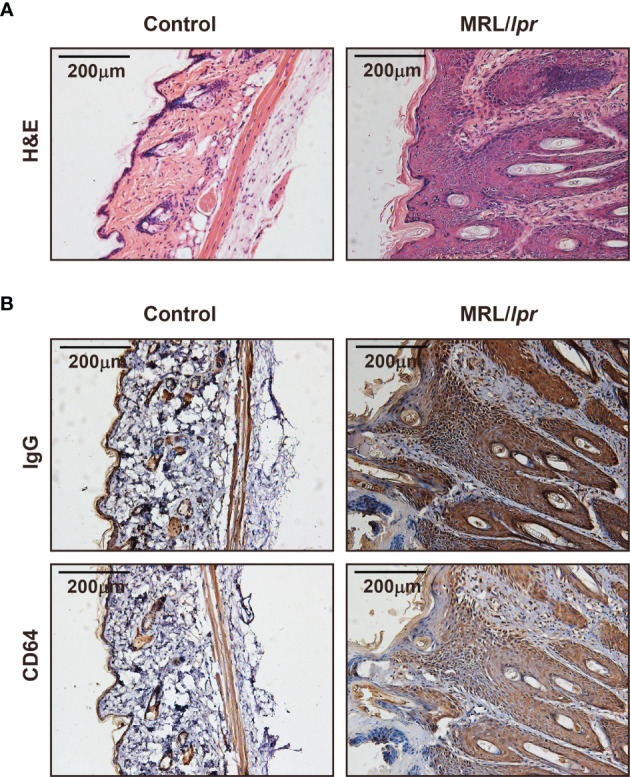
CD64 markedly increased in skin damages in MRL/*lpr* mice. **(A)** Representative photograph of H&E staining skin inflammation of female 30-week-old MRL/*lpr* mice and normal C57BL/6 mice. **(B)** Representative photograph of IgG and CD64 deposition stained by immunohistochemistry in skins of female 30-week-old MRL/*lpr* mice and normal C57BL/6 mice.

MRL/*lpr* mice could develop lupus-like clinical symptoms spontaneously, including skin inflammation. We examined IgG deposition and CD64 expression in skin lesions in MRL/*lpr* mice. Histopathology indicated a number of inflammatory cells infiltrated in the skin (**Figure 3A**). Inflammatory sites have a large amount of IgG deposited (**Figure 3B**). Furthermore, we found that CD64 expression was related to IgG deposition in skin inflammatory sites of MRL/*lpr* mice (**Figure 3B**). Based on these findings, CD64 levels are related to IgG deposition and skin inflammation.

### IL-10 and IFN-γ Increased CD64 Expression in Monocytes/Macrophages

**Figure 4:**
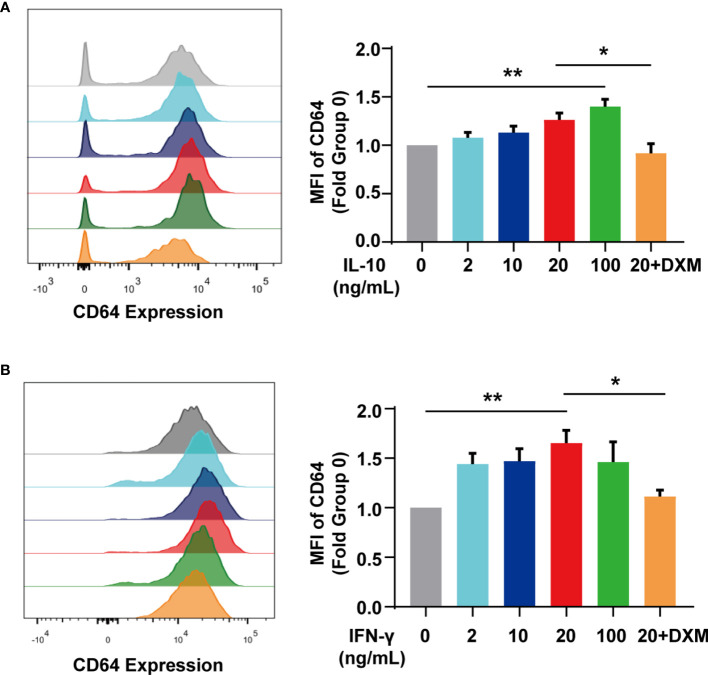
IL-10 and IFN-γ upregulated CD64 expression. **(A)** Flow cytometry detected CD64 expression in monocytes stimulated with various doses of IL-10 or 10 μM dexamethasone (DXM) for 20 h. Relative expression of CD64, showed as the fold group 0 ng/mL in MFI of monocytes. **(B)** Flow cytometry detected CD64 expression in monocytes stimulated with various doses of IFN-γ or 10 μM dexamethasone (DXM) for 20 h. Relative expression of CD64, displayed as the fold group 0 ng/mL in MFI of monocytes. *p < 0.05, **p < 0.01.

According to our previous studies, IgG in SLE serum effectively downregulated CD64 expression in monocytes (24). SLE patients, however, had higher levels of CD64 expression than controls. To investigate the potential inducer of upregulating CD64, we stimulated monocytes/macrophages isolated from mouse spleen with cytokines *in vitro* as cytokines in SLE serum are increased (**Table 2**). We found IL-10 (**Figure 4A**) and IFN-γ (**Figure 4B**) elevated CD64 expression in monocytes/macrophages, and the elevations were associated with the cytokines dose. As dexamethasone is commonly used to treat SLE patients, we investigated the effect of dexamethasone in CD64 upregulation induced by cytokines. We found that the CD64 upregulation induced by IL-10 and IFN-γ was prevented by the medication of high-dose of dexamethasone (**Figures 4A, B**). According to these findings, the therapeutic effect of glucocorticoids is mediated, at least partially, by inhibiting elevated CD64 expression induced by inflammatory cytokines.

### The Role of CD64 in Skin Inflammation Induced by Lupus Serum

**Figure 5:**
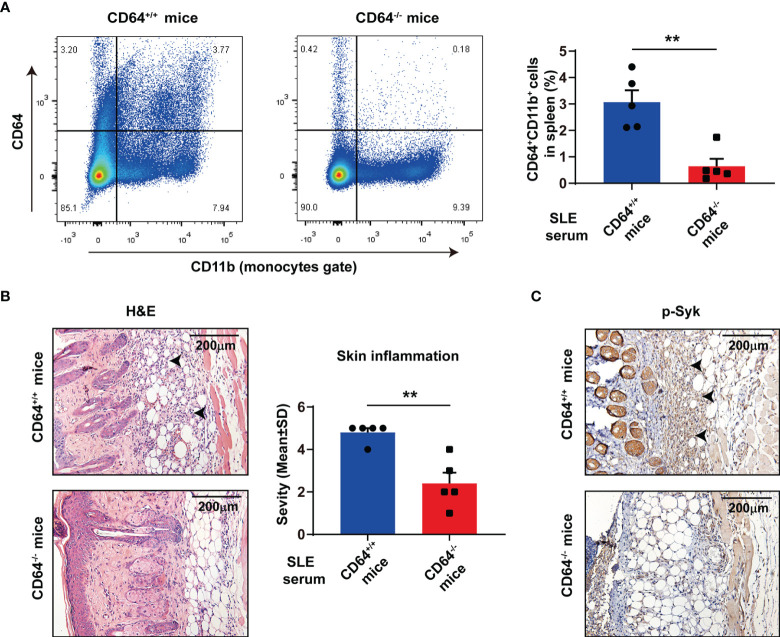
Deficiency of CD64 in mice alleviated skin inflammation induced by SLE serum. **(A)** Flow cytometry analysis of CD64 and CD11b in splenic cells. n=5 per group. **p < 0.01. **(B)** Representative histopathology of skin inflammation from CD64 deficient (^-/-^) mice and wild (^+/+^) mice with intradermal injection of 100 μL lupus serum. Black arrows refer to inflammatory cells. **(C)** Immunohistochemistry of phosphorylated Syk (p-Syk) in the skins from CD64-deficient and wild mice with intradermal injection of lupus serum. Black arrows refer to deposited p-Syk.

To understand the role of CD64 in SLE, we established skin inflammation induced by lupus serum in CD64-deficient mice. There was no noteworthy difference in weight, skin, and spleen between CD64-deficient mice and wild-type mice. Flow cytometry data established that CD64 expression was knockout in monocytes from CD64-deficient mice that we used in research (**Figure 5A**). According to histopathology, the severity of skin inflammation induced by SLE serum was significantly reduced in CD64-deficient mice compared with wild mice (**Figure 5B**). Immunohistochemistry (IHC) staining revealed that activation of Syk was decreased in the skins of CD64^-/-^ mice compared with wild mice (**Figure 5C**). The results showed that CD64 plays an important role in cutaneous injury in SLE.

### CD64 Is Required for SLE Serum-Mediated Activation of Syk, Akt, and Erk

**Figure 6:**
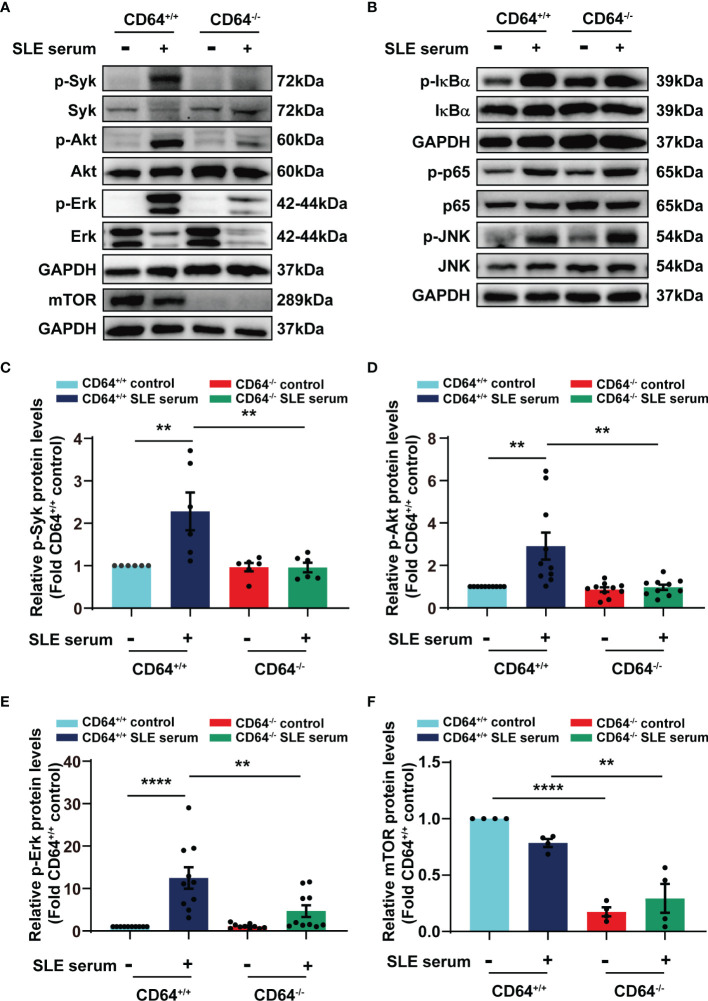
SLE serum promoted inflammation through CD64/Syk/Akt/Erk signaling pathway in macrophages. Bone marrow-derived macrophages (BMMs) were isolated from CD64 wild (^+/+^) and CD64 deficient (^-/-^) mice. Western blot identified protein levels in BMMs stimulated with 20μL SLE serum or normal saline for 2 h. **(A)** Representative picture of phosphorylated Syk (p-Syk) and total Syk, phosphorylated Akt (p-Akt) and total Akt, phosphorylated Erk (p-Erk) and total Erk, and mTOR protein levels measured by Western blot. **(B)** Western blot identified phosphorylated IκBα (p- IκBα) and total IκBα, phosphorylated p65 (p-p65) and total p65, phosphorylated JNK (p-JNK) and total JNK in BMMs. **(C–F)** Bar graphs depicting the changes in the relative expression of p-Syk **(C)**, p-Akt **(D)**, p-Erk **(E)** and mTOR **(F)**. **p < 0.01, ****p < 0.0001.

To gain insight into the molecular mechanisms by which CD64 promotes inflammation, we examined the effects of CD64 knockout on the signaling pathways activated by SLE serum. We noticed that activation of Syk, Akt, and Erk was observed at 2 h after SLE serum treatment, and the activation was decreased in CD64-deficient monocytes compared with wild monocytes (**Figures 6A**, **C**–**E**). We further detected mTOR expression in cell expressing Akt-S473 as a mTORC2 substrate. There was a clear reduction in the mTOR in the CD64 knockout monocytes, neither with SLE serum-stimulated or not, representing that mTORC2 activity was significantly reduced (**Figures 6A, F**). Alternatively, activation of NF-κB (IκBα, p65) and JNK induced by lupus serum was not inhibited in CD64-deficient monocytes (**Figure 6B**). These results suggested that SLE serum-activation of Syk, Akt, and Erk requires CD64, while activation of NF-κB and JNK is not CD64-dependent.

## Discussion

In previous studies, we demonstrated that tissue deposited lupus IgG could trigger tissue inflammation, and monocytes/macrophages, not lymphocytes or neutrophils play important roles in tissue inflammation triggered by lupus IgG (19, 23–25). As a promising new marker for bacterial sepsis, CD64 expression on neutrophils and monocytes had been linked with sepsis of critically ill neonates and children (26). The present study indicates that expression of CD64 but not CD32 and CD16 on monocytes is upregulated in SLE compared with healthy controls. In line with our observations, several studies had showed that expression of CD64 on circulating monocytes was in parallel with the serum immune complex level (27), ongoing inflammation and nephritis (28), and type-I interferon levels (29) in SLE. Our data show that the expression of CD64 on monocytes is associated with the SLEDAI, blood urea nitrogen levels, and anti-Sm antibodies in SLE patients, which indicate CD64 might be linked with the activity of diseases and organ damages.

The cytokine IL-10, which is anti-inflammatory and tolerogenic, promotes B cell responses and is pathogenic in SLE (30, 31). IFN- γ is one of the significant cytokines that stimulate monocytes to switch their differentiation from dendritic cells to CD14^-^CD64^+^ macrophages (32). In our study, both IL-10 and IFN-γ raise the expression of CD64 on monocytes/macrophages in a dose-dependent manner. Glucocorticoids block the effects of IL10 and IFN-γ on CD64 expression. Similar results had been reported with both cytokines upregulated CD64 surface expression in autologous monocytes; IL-10, but not IFN-γ failed to induce CD64 elevation in human neutrophils (33). Glucocorticoids may prevent the production of IL-10 and IFN-γ (34). Therefore, glucocorticoids, as powerful therapeutic agents in SLE, are partly due to direct effects on cytokines like IL-10 and IFN-γ on CD64 expression. Nevertheless, it is unclear for us why levels of cytokines IL-10 and IFN-γ in SLE sera have no significant difference between high and low CD64 group patients.

There are large amounts of IgG and CD64 in skin lesions of MRL/*lpr* mice, and skin inflammation caused by lupus serum was reduced significantly in CD64-deficient mice. We presented that Syk inhibition suppresses the growth of lupus skin and kidney disease in lupus-prone mice (35). Syk mediated skin inflammation by activating Src family downstream (36). The inhibition of Syk reduced skin inflammation and improved epidermal barriers *in vivo* with atopic dermatitis (37). Variants of Syk were identified in patients with multiorgan inflammation accompanied by increased activation and increased downstream signaling (38). Especially, the outcomes here indicated that the expression of phosphorylated Syk was reduced in sites of lupus serum injection in CD64-deficient mice, showing alleviated skin inflammation.

We provide mechanistic insights into SLE with our studies. As we demonstrated previously, the most prominent component of lupus serum IgG-mediated inflammation is monocytes/macrophages (20–22). Lupus serum and its IgG activated Syk and NF-κB signaling, resulting in the release of TNF-α (19, 23). Herein, SLE serum also activates Akt and MAPKs (Erk and JNK) signaling. Consistently, Akt signaling and MAPKs signaling have been proved to be important in skin inflammation (15, 39–43). According to studies, ultraviolet B causes acute skin inflammation by phosphorylating MAPKs and Akt in human keratinocytes (39, 40). Likewise, amelioration of skin inflammation is achieved through inhibition of MAPK and Akt signaling (15). Funding et al. stated that oxazolone-induced skin inflammation is reduced in MAPK AP kinase 2 knockout mice (41). The inhibition of MAPK pathways is a significant step for glutamine and fish scale collagen peptides reducing skin inflammation (42, 43). Additionally, the reduction of phosphorylated Akt-S473 in the CD64 knockout mice represents the inhibition of mTORC2. The mTOR protein, with two complexes, mTORC1 and mTORC2, acts as a regulator of cell growth, metabolism, and diseases (44). Through Akt activation, mTORC2 increases mTORC1, which enhances protein synthesis and inhibits autophagy (45). The mTOR blockade with sirolimus (rapamycin) increases the T cell-mediated immunity and improves the diseases activity in SLE patients (46). mTOR-dependent lineage differentiation regulates inflammation in multiple ways, and mTOR blockade may be a therapeutic target (45). Here, the deficiency of CD64 obviously inhibits the activation of Syk, Akt, Erk and significantly reduces mTOR, suggesting that CD64-mediated skin inflammation is dependent on Syk, Akt, Erk, and mTOR.

NF-κB signaling controls the cell biology process in survival, inflammation, and other responses (47). However, the role of NF-κB activation in skin inflammation seems to be debatable. Although it has been reported that the epidermis-specific deletion IκB kinase 2 inhibits NF-κB activation, severe skin inflammation developed (48). Here, activation of NF-κB (p65 and IκBα) is still detected in monocytes/macrophages with deletion of CD64 induced by SLE serum, but skin inflammation is alleviated in CD64-deficient mice. It is thus crucial to understand why both enhanced and defective NF-κB signaling is able to cause skin inflammation.

There are a few limitations to be considered in this study. First, SLE patients with infectious diseases had not been excluded or categorized. As already stated, CD64 is also associated with bacterial infections or other pathogens. Second, CD64 expression in monocytes can be altered by glucocorticoids, while some of the patients had been treated with glucocorticoids before. Third, we studied a comparatively small number of SLE patients and healthy controls for a single data.

In conclusion, the current study has demonstrated that upregulation of surface CD64 expression in monocytes is related to elevated SLEDAI, blood urea nitrogen levels, and anti-Sm antibodies in SLE patients; IL-10 and IFN-γ elevate CD64 expression which can be eliminated by glucocorticoids. The deficiency of CD64 reduced lupus serum-induced skin inflammation and inhibited the activation of Syk, Akt, and Erk. These data suggest that monocytes/macrophages surface CD64 measurement might be a useful tool for diagnosing SLE, and specific blockade CD64 might signify a therapeutic target for organ tissue damage in SLE.

## Materials and Methods

### Patients and Controls

SLE patients were selected satisfying four or more of the revised 1997 American College of Rheumatology (ACR) (49) in Wuhan Union Hospital from January 2021 to December 2021. The study included 45 SLE patients and 18 healthy controls. In **Table 1**, demographic data (age and gender), clinical manifestations, laboratory measurements and medication usage were summarized. SLE patients were further categorized into low CD64 (MFI<13763, n=22) and high CD64 group (MFI≥13763, n=23) according to a median of MFI. Their laboratory measurements and dose of glucocorticoids were presented in **Table 2**. Informed consent was received from all participants under the Wuhan Union Hospital Review Board-approved protocol (Number: 0267-01).

### Mice

CD64-deficient (18033) model organisms were obtained from Shanghai Model Organisms Center. C57BL/6 mice were procured from the Animal Center of Huazhong University of Science and Technology (HUST). MRL/*lpr* mice (000485) were procured from Jackson Laboratory (Bar Harbor, ME). All mice were kept and fed under standard pathogen-free environments with a 12 h light/dark cycle. The animal experiments protocol was approved by the Institutional Animal Care and Use Committee of HUST (IACUC Number: 2484).

### Flow Cytometry

We collected heparinized whole blood (2mL) from SLE patients and controls and isolated PBMCs. In 100 mL PBS, 2×10^6^ PBMCs were suspended. The cells were stained with allophycocyanin (APC)-conjugated anti-CD64 (10.1, BD), APC-conjugated anti-CD32 (FLI8.26, BD), APC-conjugated anti-CD16 (B73.1, BD). Cells were simultaneously stained with phycoerythrin (PE)-conjugated CD14 (M5E2, Biolegend) to characterize monocytes for 30 min in the dark. Mouse spleen cells were collected after lysis buffer was used to remove red cells from one half of the spleen. The cells were stained with PE-conjugated anti-CD64 (X54-5/7.1, BD) and APC-conjugated anti-CD11b (M1/70, Biolegend); 3×10^5^ cells were analyzed by flow cytometer (BD). For monocytes/macrophages cultured and stimulated by IL-10, IFN-γ and dexamethasone (DXM) *in vitro*, 1×10^4^ cells were analyzed by flow cytometer. Based on monocytes’ forward/sideward light scatter patterns and their expression of CD14, gates were set around them. All the flow cytometry data were analyzed using FlowJo 10 (TreeStar, USA).

### Isolation and Culture of Bone Marrow-Derived Macrophages and Splenic Monocytes/Macrophages

To obtain bone marrow-derived macrophages (BMMs), 6-week-old C57BL/6 and CD64 deficient male mice were sacrificed by cervical dislocation. The bone marrow cells (BMCs) were isolated from the tibia and femur by instant high-speed centrifugation, and then cells were cultured for 3 h with a serum-free medium. We removed the adherent cells and cultured the non-adherent BMCs in suspension with α-MEM medium with 10% FBS, 1% penicillin-streptomycin, and 30 ng/mL of M-CSF for 6 d, and the medium was changed every 3 d. Then the BMMs were harvested and stimulated using 20 μL SLE serum for 2 h. Mouse spleens were ground, and mononuclear cells were isolated to obtain splenic monocytes/macrophages. Monocytes were obtained after suspended lymphocytes were removed by incubation at 37°C for 3 h. We added M-CSF (10 ng) to the plates and cultured them for 3 d. Then several dose (0 ng/mL, 2 ng/mL, 10 ng/mL, 20 ng/mL, 100 ng/mL) of IL-10 and IFN-γ, and 10 μM DXM were added and stimulated monocytes/macrophages for 20 h.

### Injection Protocol, Histopathology and Immunohistochemistry (IHC)

8-week-old female C57BL/6 and CD64-deficient mice were intradermally injected 100 μL SLE serum to induce skin inflammation. Then skins were fixed in 4% paraformaldehyde three days after injection. After fixation, the samples were dehydrated in ethanol, embedded in paraffin, cut into 5 mm pieces, and stained with hematoxylin and eosin (HE). Skin inflammation was evaluated as our previous publication (18). The tissue slides were incubated overnight with an anti-phospho-Syk (TA8404, Abmart) antibody at 4°C for the IHC assay. Afterwards, slides were incubated with biotinylated secondary antibodies, and all sections were counterstained with Mayer’s hematoxylin.

### Western Blotting

Immunoblotting experiments were conducted with whole-cell were lysed in radio immunoprecipitation assay (RIPA) buffer. Protease inhibitors and phosphatase inhibitors were added in RIPA buffer to avoid protein degradation. After eliminating cell debris, cell lysates were boiled for 5 min with SDS loading buffer, and resolved on SDS-PAGE gels. The proteins were then transferred onto polyvinylidene fluoride membranes (Millipore, USA) by using the Trans-Blot^®^ Turbo™ Blotting System (Bio-Rad). Afterwards, the membranes were blocked with 5% bovine serum albumin (BSA) and further incubated with the indicated antibodies. Anti-phospho-Syk (AP0524), anti-Syk (A2123), anti-phospho-p65 (AP0123), anti-p65 (A19653) and anti-GAPDH (AC002) were procured from ABclonal. Anti-phospho-Akt (56569), anti-Akt (55561), anti-phospho-Erk (57165), and Erk (55487), anti-phospho-IκBα (56280), anti-IκBα (55026), anti-phospho-JNK (56315), and JNK (40073) were obtained from Abmart. Anti-mTOR (4517) was obtained was acquired from CST. For protein detection, ChemiDoc Touch Imaging System from Bio-Rad was used.

### Statistical Analysis

All the results, including at least three independent experiments, are presented as mean ± SEM. Data were analyzed by unpaired two-tailed Student’s t-test (for two groups) or one-way ANOVA test (for ≥3 groups). GraphPad Prism 8 (GraphPad Software, Inc.) was used to execute all statistical analyses.

## Data Availability Statement

The original contributions presented in the study are included in the article/supplementary material. Further inquiries can be directed to the corresponding author.

## Ethics Statement 

The studies involving human participants were reviewed and approved by Wuhan Union Hospital Review Board. The patients/participants provided their written informed consent to participate in this study. The animal study was reviewed and approved by Institutional Animal Care and Use Committee (IACUC) guidelines of Huazhong University of Science and Technology.

## Author Contributions

G-MD and LJ: Design of research plan and experiments; analysis of results; writing of the manuscript. LJ: Conduction of major experiments; analysis of results. XH, WQ, TY, RF, XW, and XD: Contribution of data for the manuscript. All authors contributed to the article and approved the submitted version.

## Funding

This study was supported by Free Research Fund (GM Deng, 02.03.2019-135) and Research Initiating Fund (GM Deng, 02.03.2018-41) of Union Hospital, Tongji Medical College, Huazhong University of Science and Technology, Wuhan, China.

## Conflict of Interest

The authors declare that the research was conducted in the absence of any commercial or financial relationships that could be construed as a potential conflict of interest.

## Publisher’s Note

All claims expressed in this article are solely those of the authors and do not necessarily represent those of their affiliated organizations, or those of the publisher, the editors and the reviewers. Any product that may be evaluated in this article, or claim that may be made by its manufacturer, is not guaranteed or endorsed by the publisher.
